# Use of ethanol extracts of *Terminalia chebula* to prevent periodontal disease induced by dental plaque bacteria

**DOI:** 10.1186/s12906-017-1619-1

**Published:** 2017-02-16

**Authors:** Jongsung Lee, Youn Hwa Nho, Seok Kyun Yun, Young Sun Hwang

**Affiliations:** 1 Department of Genetic Engineering, Sungkyunkwan University, Suwon, 164-19 Republic of Korea; 2 COSMAX R&I Center, COSMAX Inc., Seongnam, 134-86 Republic of Korea; 3 Department of Dental Hygiene, College of Health Science, Eulji University, 553, Sansung-Daero, Soojung-Gu, Seongnam City, Republic of Korea

**Keywords:** Ethanol extract of *Terminalia chebula* (EETC), Gingivitis, Periodontitis, Dental plaque bacteria (DPB), Lipopolysaccharide (LPS), Inflammation, Osteoclast

## Abstract

**Background:**

The fruit of the *Terminalia chebula* tree has been widely used for the treatment of various disorders. Its anti-diabetic, anti-mutagenic, anti-oxidant, anti-bacterial, anti-fungal, and anti-viral effects have been studied. Dental plaque bacteria (DPB) are intimately associated with gingivitis and periodontitis. In the quest for materials that will prove useful in the treatment and prevention of periodontal disease, we investigated the preventive effects of an ethanol extract of *Terminalia chebula* (EETC) on DPB-induced inflammation and bone resorption.

**Methods:**

The anti-bacterial effect of EETC was analyzed using the disc diffusion method. The anti-inflammatory effect of EETC was determined by molecular biological analysis of the DPB-mediated culture cells. Prevention of osteoclastic bone resorption by EETC was explored using osteoclast formation and pit formation assays.

**Results:**

EETC suppressed the growth of oral bacteria and reduced the induction of inflammatory cytokines and proteases, abolishing the expression of PGE2 and COX-2 and inhibiting matrix damage. By stimulating the DPB-derived lipopolysaccharides, EETC inhibited both osteoclast formation in osteoclast precursors and RANKL expression in osteoblasts, thereby contributing to the prevention of bone resorption.

**Conclusions:**

EETC may be a beneficial supplement to help prevent DPB-mediated periodontal disease.

## Background

The oral cavity is a suitable milieu for bacterial growth and propagation. The presence of bacteria in the mouth readily stimulates the formation of dental plaque, which accumulates on both hard and soft tissues as dental calculus. Although the regional colonization and invasion of bacteria are rigorously controlled by the dynamic equilibrium between dental plaque bacteria (DPB) and the host’s innate defense mechanisms [1], plaque that extends subgingivally can trigger the immune system imbalance, inducing an inflammatory response [2]. Gingivitis and periodontitis are the most common plaque-induced inflammatory conditions. *Aggregatibacter actinomycetemcomitans*, *Porphyromonas gingivalis*, *Prevotella intermedia*, and *Tannerella forsythensis* are the most prevalent anaerobic gram-negative bacteria in subgingival area. All are critical in the onset and subsequent development of periodontitis. If untreated, these bacteria can lead to the periodontal pocket, connective tissue destruction, and alveolar bone resorption [3].

Bacteria involved in the initiation and progression of periodontal disease are classified into color-coded groups. The categories are based upon the pathogenicity of the bacteria and their role in the development of plaque [4]. Species in the red complex (*P. gingivalis*, *T. forsythia*, and *Treponema denticola*) are strongly related with pocket depth and bleeding on probing. The red complex can be used as a clinical parameters that is considered the most meaningful in periodontal diagnosis. Species in the orange complex (*Fusobacterium nucleatum*, *P. intermedia*, *Prevotella nigrescens*, *Peptostreptococcus micros*, *Streptococcus constellatus*, *Eubacterium nodatum*, and *Campylobacter gracilis*) are closely associated with one another. The orange complex is closely related to the red complex. Similarly to the red complex, the orange complex is significantly associated with increasing pocket depth; a decreased level of these species lead to improvement in periodontal status. *A. actinomycetemcomitans*, which is classified in the green complex, is associated with localized aggressive periodontitis.

Toxins produced by DPB, such as lipopolysaccharide (LPS), stimulate an inflammatory response. LPS induces the release of various inflammatory mediators from gingival tissue, which activates the innate immune system of the host to eliminate the inflammation [5]. Although such mediators are normally part of a beneficial host response to suppress bacterial proliferation, they can also damage the area of inflammation. During the inflammatory-immune response in gingivitis and periodontitis, inflammatory cytokines and matrix metalloproteinases (MMPs) are produced, which destroy the extracellular matrix (ECM) and lead to periodontal bone loss [6]. In periodontal disease, the irreversible breakdown of collagen fibers in the periodontal ligament results in tissue destruction.

Epidemiological studies have consistently revealed the increase risk for periodontal disease with age. The National Health and Nutrition Examination Survey (NHANES) recently estimated that in the United States 47.2% of adults 30 years of age and older have periodontitis [7], and the prevalence of periodontitis among all adults increases with increasing age. The fact that over the past 2 years the risk of periodontitis has increased approximately 53.0% indicates the need for more intensive oral hygiene and dental care, and the dissemination of information regarding the treatment and prevention of periodontal disease [8].

The properties of various extracts of the fruit of the *Terminalia chebula* tree have been widely investigated and include anti-diabetic, anti-mutagenic, anti-oxidant, anti-bacterial, anti-fungal, and anti-viral effects [9]. Many of these beneficial effects are related to the presence of various phytochemicals including polyphenols, terpenes, anthocyanins, flavonoids, alkaloids, and glycosides [10]. In the present study, we determined the effects of an ethanol extract of *T. chebula* (EETC) in preventing DPB-induced inflammation and bone resorption, and identified the principal molecules in this inflammatory response that are regulated by EETC. The data indicate the potential value of EETC in preventing DPB-mediated periodontal disease.

## Methods

### Materials and reagents

Minimum essential medium alpha medium (α-MEM), RPMI 1640 medium, Dulbecco’s modified Eagle’s medium (DMEM)/F-12 phenol red-free medium (1:1), fetal bovine serum (FBS), antibiotic-antimycotic mixture (100×), phosphate-buffered saline (PBS), and 0.25% trypsin-EDTA (1×) were purchased from Gibco BRL Co. (Grand Island, NY). Dimethyl sulfoxide (DMSO), LPS, and 3-(4,5 dimethylthiazol-2-yl)-2,5-diphenyltetrazolium bromide (MTT) were obtained from Sigma-Aldrich (St Louis, MO). Recombinant mouse soluble RANK ligand (sRANKL) was purchased from Koma Biotech (Seoul, Republic of Korea). Recombinant mouse macrophage colony-stimulating factor (M-CSF) was purchased from R&D System (Minneapolis, MN). EETC was provided by COSMAX Inc. R&I Center (Seongnam City, Republic of Korea).

### Plant material

*T. chebula* fruit were collected from southwest China (Yunnan province) in 2014. Taxonomic identification was done by a botanist and herbalist at COSMAX. A voucher specimen (CH209) was deposited in the COSMAX Inc. R&I Center.

### Extraction procedure

*T. chebula* fruit were thoroughly washed with distilled water to remove dirt and soil, and dried under shade and ventilation. The dried fruits were ground using an electronic miller. The powder was extracted using 70% ethanol for 72 h at room temperature, filtered through Whatman filter paper No. 1, and concentrated using a rotary evaporator under reduced pressure. The dried extracts were stored in a refrigerator until for further use. Stock solution was aliquoted and stored frozen at −70 °C for up to 6 months. Freeze/thaw cycles were avoided.

### Bacterial culture and preparation

*Streptococcus mutans*, *Aggregatibacter actinomycetemcomitans*, and DPB were cultured in brain-heart infusion (BHI) broth (Becton, Dickinson and Company, Baltimore, MD) at 37 °C with shaking at 200 rpm. Dental plaques were obtained through regular scaling from a participant without any oral mucosal disease and all procedures were performed by dental hygienist. Informed consent was given by the participant. Cells were harvested and PBS-washed three times by centrifugation at 3,000 rpm for 5 min. The washed cells were suspended in PBS and absorbance was measured at 600 nm using a DU 800 spectrophotometer (Beckman Coulter, Palo Alto, CA). A standard curves was generated for each bacterial sample and colony forming units (CFU)/ml was determined. For experiments requiring plate cultures, a bacterial suspension was subcultured onto the surface of BHI agar and bacterial growth was assessed. To stimulate the cultured cells, a multiplicity of infection (MOI) of 200 bacteria was applied for 24 h at 37 °C and 5% CO_2_.

#### Minimum inhibitory concentrations (MICs) assay

MICs were determined for the EETC by the broth dilution method after incubation at 37 °C for 24 h under 5% CO_2_, with each determination done in triplicate. A MIC was the lowest concentration of test sample that completely inhibited visible growth in the broth.

### LPS extraction

LPS was extracted using a LPS extraction kit (iNtRON Biotechnology, Seongnam City, Republic of Korea) according to the manufacturer’s protocol. DPBs samples (DPB^#^1, DPB^#^2, or DPB^#^3) were harvested when the culture was at an optical density at 600 nm (OD_600_) of 0.8 to 1.2. Lysis and purification buffers were sequentially applied, and the LPS pellet was dissolved by boiling for 3 min. LPS was quantified using the Limulus Amebocyte Lysate Chromogenic Endotoxin Quantitation Kit (Pierce Biotechnology, Rockford, IL) according to the manufacturer’s protocol. *Escherichia coli* O111:B4 was used as the standard of known concentration. Ten endotoxin units (EU)/mL equaled approximately 1 ng/ml.

### Cell lines and culture media

RAW264.7 macrophage cells were cultured in RPMI 1640 containing 10% FBS and 1% antibiotic-antimycotic mixture at 37 °C and 5% CO_2_. Human fetal osteoblastic cells (hFOB1.19; American Type Culture Collection, Manassas, VA) were cultured in DMEM/F-12 containing 10% FBS and 1% antibiotic-antimycotic mixture. Immortalized human oral keratinocytes (IHOK), immortalized human gingival fibroblasts (IGF), and YD38 human gingival epithelial cells were obtained from the Yonsei University College of Dentistry, Republic of Korea, and all were cultured in DMEM/F12 (3:1 ratio) as previous detailed [11]. Mouse bone marrow-derived macrophages (BMMs) were isolated from the tibias of 4-week-old ICR male mice using Histopaque density gradient centrifugation. BMMs were cultured in α-MEM containing 10% FBS, M-CSF (30 ng/ml), and a 1% antibiotic-antimycotic mixture.

### In vitro susceptibility test

In vitro susceptibility was assessed using the disc diffusion method. Briefly, the bacterial suspension in agarose solution was inoculated on BHI agar plates, and the gel was allowed to solidify completely at room temperature. Whatman filter discs with DMSO, ampicillin (Amp, 10 μg/disc), or EETC (5 μg, 10 μg/disc) were placed on the plates and cultured overnight at 37 °C and 5% CO_2_. Susceptibility was assessed using linear fitting of the squared radius (diameter in mm) of the inhibition zones. Ampicillin was used for comparing the assay results. DMSO (0.01%) was used as the control.

### Cytotoxicity assay

Cells (1 × 10^4^ cells/well) were added to wells of a 96-well culture plate. After 24 h, the fluid was replaced with complete medium containing EETC and incubation continued for 24 h. Cell viability was measured by the MTT assay, which is based on the ability of viable cells to convert soluble MTT into an insoluble dark blue formazan. MTT solution (5 mg/ml) was added to each well and the plate was incubated at 37 °C for 4 h. The purple formazan product was dissolved with 200 μl DMSO and the optical density at 570 nm was determined with a microplate reader (Bio-Rad, Hercules, CA).

### Prostaglandin E2 (PGE2) assay

Concentration of human PGE2 was measured by an ELISA kit (R&D Systems) according to the manufacturer’s protocol. Cells (1 × 10^5^ cells/well) were cultured in complete medium in wells of a 6-well plate. After being washed with PBS, the cells were exposed to serum-free medium containing LPS (1 μg/ml) and/or EETC (10 mg/ml) for 24 h. The culture medium was collected by centrifugation at 1,500 rpm for 10 min and was examined by ELISA. DPB^#^1, DPB^#^2, and DPB^#^3 were independently co-cultured with RAW264.7 macrophages, IHOK keratinocytes, IGF gingival fibroblasts, YD38 gingival epithelial cells. Cultured media were harvested by centrifugation and used to determine the level of PGE2. LPS extracted from the three DPB samples (DPB^#^1-LPS, DPB^#^2-LPS, and DPB^#^3-LPS) were also treated instead of DPB.

### Reverse transcription-polymerase chain reaction (RT-PCR)

Total RNA was isolated using the Trizol (Invitrogen, Carlsbad, CA). Single-stranded cDNA was transcribed from the RNA using Promega’s reverse transcription system (Madison, WI). Then PCR was performed with cDNA in a reaction mixture containing 25 mM magnesium chloride (MgCl_2_), dNTPs, reverse and forward primers, and *Taq* polymerase (Takara Bio, Shiga, Japan). The following primers were used for PCR: F-COX2, 5′-ATGACTTCCAAGCTGGCCGT-3′ and R-COX2, 5′-CCTCTTCAAAAACTTCTCCACACC-3′ (annealing temperature; 57 °C, PCR product size; 305 base pair): F-RANKL, 5′-GCCAGTGGGAGATGTTAG-3′ and R-RANKL, 5′-TTAGCTGCAAGTTTTCCC-3′ (annealing temperature; 48 °C, PCR product size; 486 base pair): F-OPG, 5′-GGGGACCACAATGAACAAGTTG-3′ and R-OPG, 5′-AGCTTGCACCACTCCAAATCC-3′ (annealing temperature; 55 °C, PCR product size; 409 base pair): F-GAPDH, 5′-CCCCCTACTGCCCACTGCCACCAC-3′ and R-GAPDH, 5′-TCCATCCACTATGTCAGCAGGTCC-3′ (annealing temperature; 52 °C, PCR product size; 420 base pair). The amplified PCR product was electrophoresed on a 2% agarose gel in 1× Tris-Borate-EDTA buffer containing ethidium bromide and visualized using Quantity One software and the Gel Doc 2000 system (Bio-Rad).

### Inflammation antibody array

Inflammatory cytokine profiles were measured using a Human Inflammation Array C3 Kit (RayBiotech, Norcross, GA) according to the manufacturer’s protocol. Briefly, DPB-LPS (1 μg/ml) and/or EETC (10 mg/ml) was added in culture medium and harvested after 24 h treatment for conditioned medium (CM). Cytokine array membranes were blocked with blocking buffer and incubated with equal protein amounts of CM for 24 h. After extensive sequential washing with Buffer I and Buffer II, the membrane was reacted with a cocktail of biotin-conjugated antibodies against different individual cytokines. The membrane were washed again and reacted with horseradish peroxidase (HRP)-conjugated streptavidin. Relative protein expression was detected by the enhanced chemiluminescence (ECL) system. Densitometric values were quantified using TINA-program software.

### Protease array

Before application to the array, the protein concentration of the CM was normalized by dilution with serum-free media. Then CM was incubated for 24 h with the Proteome Profiler Human Protease Array Kit (R&D Systems). The relative expression levels of the proteases were determined according to the manufacturer’s protocol, and signal intensities were compared using Quantity One software and the Gel Doc 2000 system (Bio-Rad).

### ECM degradation

Fluorescein isothiocyanate (FITC)-conjugated gelatin matrix-coated coverslips were prepared as described previously [12]. After the coverslips were quenched for 1 h with complete media at 37 °C, the cells were plated and cultured in medium with DPB-LPS (1 μg/ml) and/or EETC (10 mg/ml). Cells were fixed with 4% paraformaldehyde followed by permeabilization with 0.5% Triton X-100/PBS. Dark areas lacking fluorescence on the coverslips were observed with a LSM 510 META confocal laser scanning microscope (Carl Zeiss, Oberkochen, Germany).

### Osteoclast formation

Mouse BMMs (5 × 10^4^ cells/well) were plated and cultured in α-MEM complete media containing M-CSF (30 ng/ml), sRANKL (10 ng/ml or 100 ng/ml), DPB-LPS (1 μg/ml), and/or EETC (10 μg/ml) for 7 days with replacement with fresh medium every second day. The cells were fixed with 4% paraformaldehyde for 10 min. Cells were reacted with the Acid Phosphatase, Leukocyte (TRAP) kit (Sigma-Aldrich) according to the manufacturer’s instruction. The number of TRAP-positive multinucleated cells (≥3 nuclei) were counted as osteoclasts per well. Reaction with only M-CSF was performed for control.

### Pit formation

Osteo Assay Surface Polystyrene Stripwells (Corning, Corning, NY) were used to observe pit formation as a measure of osteoclast activity. Mouse BMMs (5 × 10^4^ cells/well) were plated on stripwells and cultured in α-MEM complete media containing M-CSF (30 ng/ml), sRANKL (10 ng/ml or 100 ng/ml), DPB-LPS (1 μg/ml), and/or EETC (10 μg/ml). Cultures were fed every 2 days with fresh medium. After a 15-days incubation period, all remaining cells were lysed using 5% sodium hypochlorite solution. Images of the resorbed pits were obtained under light microscopy.

### Statistical analysis

The statistical analysis was conducted using InStat™ statistical software (GraphPad Software, San Diego, CA). Data are expressed as mean ± standard errors. Asterisks were used to graphically indicate the statistical significance. The statistical significance of differences between groups was analyzed via repeated measures of one-way ANOVA. *P* values of <0.05 were considered significant.

## Results

### Effects of EETC on growth of DPB

**Fig. 1:**
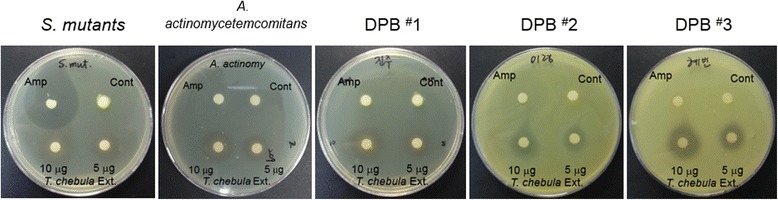
EETC suppresses bacterial growth*.* Inhibitory effect of EETC on the growth of *Streptococcus mutans* (*S. mutans*), *Aggregatibacter actinomycetemcomitans* (*A. actinomycetemcomitans*), and different types of dental plaque bacteria (DPB^#^1, DPB^#^2, and DPB^#^3) compared with ampicillin (Amp). The data shown are representative of three independent experiments

Using the disc diffusion assay, we assessed the extent to which the growth of *S. mutans*, *A. actinomycetemcomitans*, and dental plaque bacteria (DPB^#^1, DPB^#^2, and DPB^#^3) was inhibited after EETC treatment. As shown in Fig. 1, ampicillin, which has a broad spectrum of anti-bacterial activity against certain gram-positive, gram-negative, and anaerobic bacteria [13], inhibited the growth of *S. mutans* but had negligible effect on the growth of DPBs (DPB^#^1, DPB^#^2, or DPB^#^3) and *A. actinomycetemcomitans*. EETC effectively suppressed the growth of *S. mutans*, *A. actinomycetemcomitans*, and DPB, its antibacterial effect on *S. mutans* was not as pronounced as ampicillin*.* The degree of inhibition caused by EETC increased with concentration. Growth inhibition zones were measured on bacterial culture plates after treatment with ampicillin or EETC (Table 1). MIC of EETC against the DPB samples ranged from 32 to 64 μg/ml.

**Table 1:** Antibacterial effect of EETC against plaque bacteria

Compound	Concentration (μg/disk)	Size of clear zone (disk margin, mm)				
		*S. mutants*	*A. actinomycetemcomitans*	^1^DPB^#^1	^1^DPB^#^2	^1^DPB^#^3
Control	−^3^	-	-	-	-	-
Ampicillin	10	15.5 ± 0.21^b^	-	0.1 ± 0.11	-	-
^2^EETC	5	3.8 ± 0.19^b^	3.7 ± 0.03^a^	3.2 ± 0.03^a^	2.7 ± 0.09^b^	3.9 ± 0.18^b^
	10	7.2 ± 0.07^b^	7.5 ± 0.02^b^	6.9 ± 0.04^b^	5.3 ± 0.05^b^	7.4 ± 0.06^b^

^1^DPB; dental plaque bacteria, ^2^EETC; ethanol extracts of *Terminalia chebula*, ^3^No inhibition

(^a^ *P* < 0.05, ^b^ *P* < 0.01)

### Effects of EETC on DPB-induced inflammation

**Fig. 2:**
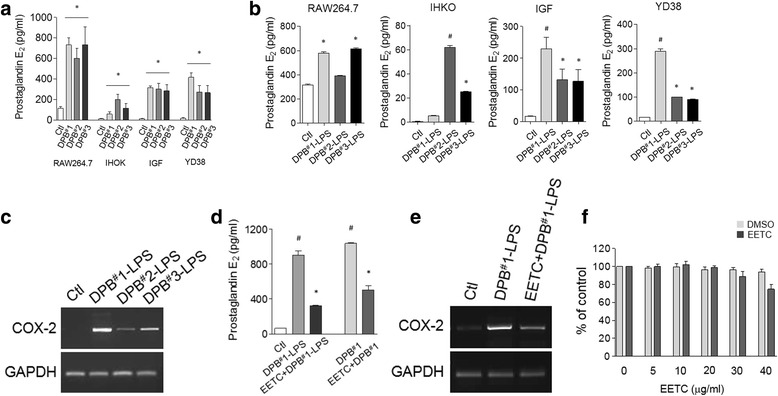
Effect of the EETC on DPB-induced PGE2 and COX-2. **a** The effect of DPB on PGE2 levels in various cell types. Data are expressed as mean ± SE of three independent experiments. *P < 0.01 vs. bacteria-free control medium from each cell line. **b** The effect of LPS extracted from DPBs (DPB^#^1-LPS, DPB^#^2-LPS, and DPB^#^3-LPS) on PGE2 levels. PBS was used for control. *P < 0.01, ^#^ *P* < 0.001 vs. PBS control medium from each cell line. **c** The effect of DPBs-LPS on COX-2 expression. mRNA expression were detected by RT-PCR. The inhibitory effects of EETC on PGE2 (**d**) and COX-2 (**e**) levels in DPB-LPS-treated cells. DMSO was used for control. Data represent the mean ± SE of three independent experiments performed by triplicate. ^#^ *P* < 0.001 vs. DMSO control (Ctl) medium, *P < 0.01 vs. DPB^#^1 or DPB^#^1-LPS medium. **f** The effect of EETC on the cell viability

PGE2, a major end product of cyclooxygenase (COX-2), has an important role in both acute and chronic inflammatory responses [14]. First, we investigated whether DPB could stimulate PGE2 expression in various cell lines. As shown in Fig. 2a, PGE2 levels were significantly increased in DPB-stimulated RAW264.7 macrophages, and IHOK, IGF, and YD38. However, the pH changes occurred slowly in co-culture media with DPB during 24 h of incubation. Therefore, we extracted LPS from DPB, and used 1 μg/ml each of DPB^#^1-LPS, DPB^#^2-LPS, and DPB^#^3-LPS for the next experiments. In RAW264.7, IHOK, IGF, and YD38 cells, PGE2 levels were also increased after DPB-LPS treatment, although the different effects were cell line-specific (Fig. 2b). COX-2 mRNA was also increased by stimulation with DPB^#^1-LPS, DPB^#^2-LPS, or DPB^#^3-LPS (Fig. 2c). Treatment with EETC significantly suppressed the levels of PGE2 and COX-2 that had been increased by DPB-LPS (10 μg/ml) (Fig. 2d and e). YD38 cell viability remained unchanged after treatment with 5 and 30 μg/ml EETC (Fig. 2f). Thus, the EETC inhibited not only the growth of DPB through its anti-bacterial effect but also inflammation by abolishing PGE2 and COX-2 expression, thereby enhancing its preventive effect on gingival and periodontal diseases.

### Elucidation of molecules involved in DPB-induced inflammation

**Fig. 3:**
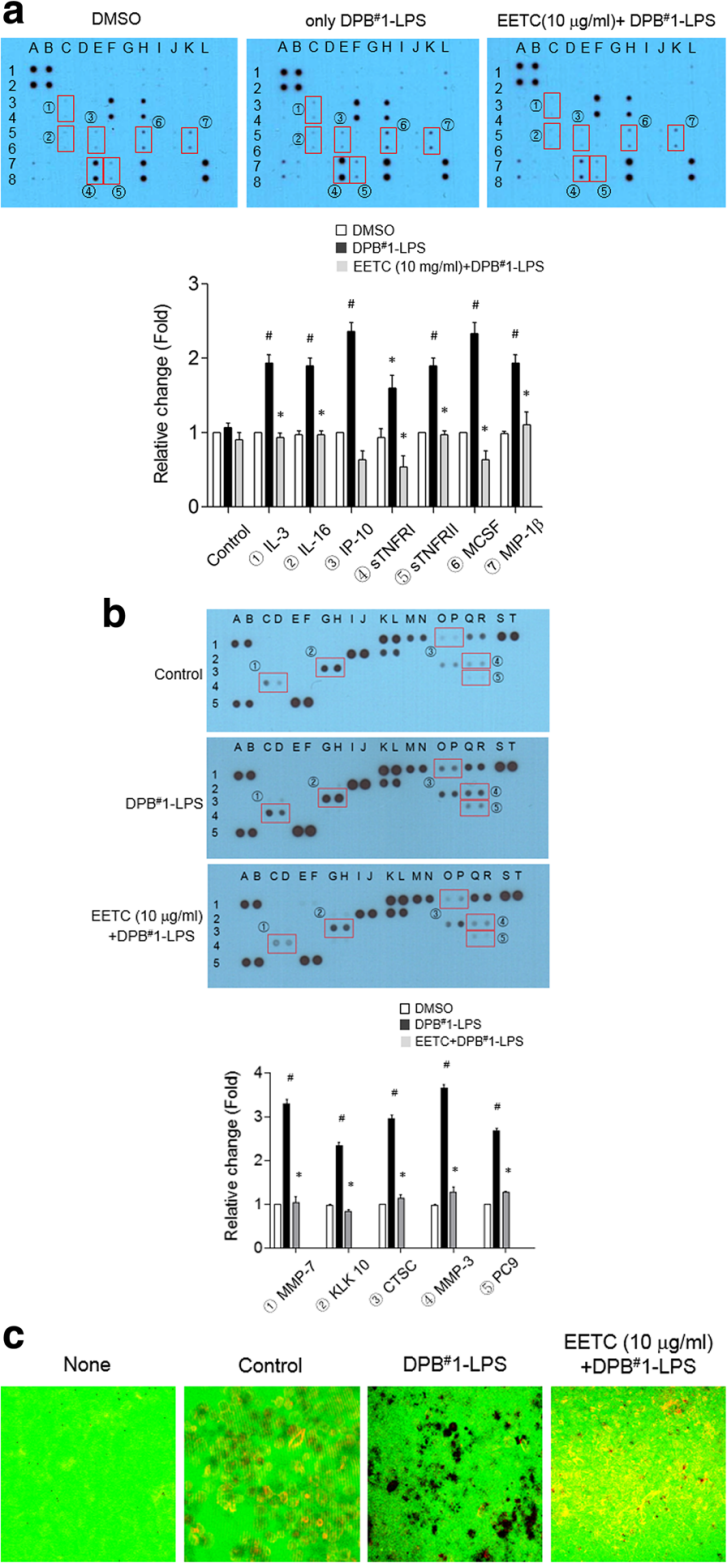
Effect of EETC on DPB-induced molecules involve in inflammation and tissue damage. **a** Inflammatory cytokine modulated by DPB-LPS and EETC in gingival epithelial cell. Analysis of culture medium (CM) collected from DMSO-treated cells, which served as the control. Altered factors are indicated with rectangles and circled numbers. The list altered factor is shown as fold-change in the graph. The data shown are representative of three independent experiments. *P < 0.01 vs. DPB1^#^-LPS-treated medium, ^#^ *P* < 0.001 vs. DMSO control medium. **b** Protease modulated by DPB-LPS and EETC in gingival epithelial cell. Altered factors are indicated with rectangles and circled numbers. The list altered factor is depicted in the graph and is shown as fold-change. The data shown are representative of three independent experiments. *P < 0.01 vs. DPB1^#^-LPS-treated medium, ^#^ *P* < 0.001 vs. DMSO control medium. **c** Effect of tissue damage by DPB-LPS and/or EETC on FITC-conjugated gelatin matrix-coated coverslips. The data shown are representative of five independent experiments

The Human Inflammation Antibody Array was used to elucidate the molecules related to the DPB-induced inflammation. In the DPB-LPS–stimulated YD38 cells, the inflammatory cytokine profiles were increased for interleukin (IL)-3, IL-16, CXC motif chemokine 10 (CXCL10, IP-10), tumor necrosis factor receptor superfamily member 1A (TNFRSF1A, TNFRI), tumor necrosis factor receptor superfamily member 1B (TNFRSF1B, TNFRII), macrophage colony-stimulating factor 1 (CSF 1, MCSF), and CC motif chemokine 4 (CCL4, MIP-1β) (Fig. 3a). Increased levels of inflammation-related molecules were suppressed by pretreatment with the EETC. In addition, as shown in human protease profiles, proteases that act on inflammation through the regulation of pro-inflammatory cytokines, chemokines, and other proteins were also increased in the DPB-LPS–stimulated YD38 cells (Fig. 3b). The levels of MMP-7 (matrilysin, PUMP-1), KLK 10 (kallikrein 10), CTSC (cathepsin C), MMP-3 (stromelysin-1), and PC9 (proprotein convertase 9) were significantly increased; the induced levels of proteases were also suppressed by pretreatment with the EETC. To validate the tissue damage due to DPB-LPS, YD38 cells were cultured on FITC gelatin-coated coverslips, and matrix degradation was observed by bleaching the fluorescence dye. As shown in Fig. 3c, DPB-LPS stimulated matrix degradation, and the EETC effectively inhibited the DPB-LPS–induced matrix degradation.

### Effects of EETC on DPB-LPS-induced bone resorption

**Fig. 4:**
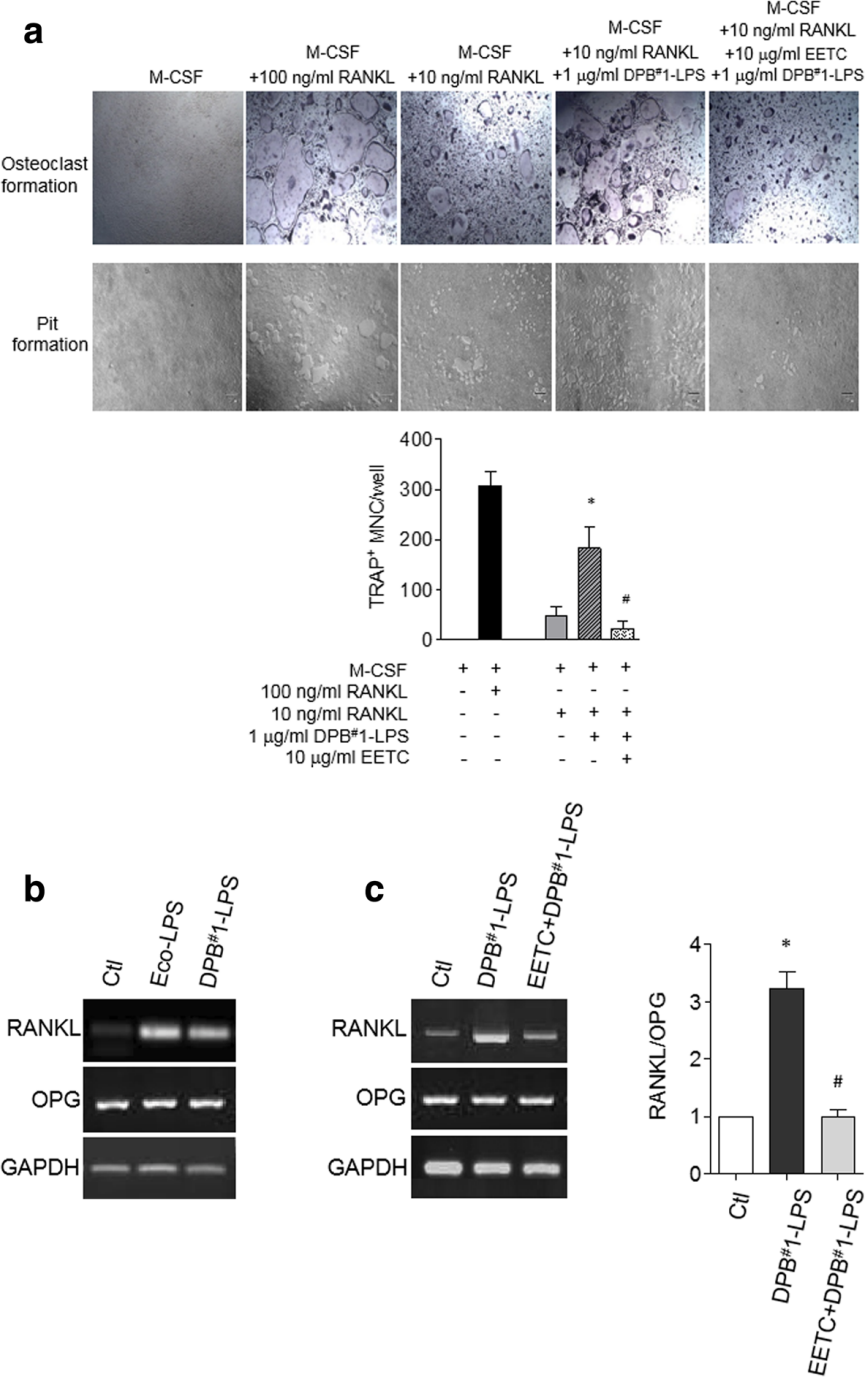
Effect of EETC on DPB-induced osteoclastic bone resorption. **a** For osteoclast formation, mouse BMMs were treated with M-CSF and RANKL and were stained to detect expression of TRAP. Pit formation was also performed in M-CSF and RANKL on calcium phosphate apatite-coated plates, and light microscopy indicated resorptive pitting. To observe the stimulating effect of DPB-LPS on osteoclast formation and pit formation, both assays were performed at a low concentration of RANKL (10 ng/ml). The graph shows the total number of TRAP-positive multinucleated (≥3 nuclei) osteoclasts (MNC) per well. Data are expressed as mean ± SE of three independent experiments. *P < 0.01 vs. only 10 ng/ml RANKL condition, ^#^ *P* < 0.001 vs. DPB1^#^-LPS plus 10 ng/ml RANKL condition. **b** RANKL and OPG mRNA expression was analyzed in hFOB1.19 human osteoblastic cells. Eco-LPS (LPS from *Escherichia coli*) was the positive control. The data shown are representative of three independent experiments. **c** Effect of EETC on DPB^#^1-LPS-induced RANKL and OPG mRNA expression were analyzed by RT-PCR. The ratio of RANKL to OPG was determined after normalization to the intensity of GAPDH. The data shown are representative of three independent experiments. *P < 0.001 vs. control (Ctl), ^#^ *P* < 0.001 vs. DPB^#^1-LPS condition

RANKL is a critical molecule in osteoclast differentiation and bone resorption. As shown in Fig. 4a, osteoclast formation was clearly detected in medium containing 100 ng/ml of RANKL, whereas TRAP-positive osteoclasts were not detected in the absence of RANKL. In order to observe the effect of DPB-LPS on osteoclast formation, we performed the reaction in media with low levels of RANKL (10 ng/ml), and DPB-LPS was added to cultures of isolated BMMs. The number of TRAP-positive osteoclasts was significantly reduced in response to 10 ng/ml RANKL, as compared with 100 ng/ml RANKL; however, osteoclast formation in 10 ng/ml RANKL condition was significantly stimulated by DPB-LPS treatment. The stimulating effect of DPB-LPS was inhibited by the EETC treatment. The bone resorption activity of mature osteoclasts was measured simultaneously. Consistent with the osteoclast formation studies, the formation of resorption pits was remarkably increased by DPB-LPS treatment and suppressed by the EETC treatment. In addition, the mRNA expression of RANKL was significantly stimulated by DPB-LPS treatment in the hFOB1.19 human osteoblast cells but remained the same for osteopontin (OPG) (Fig. 4b). Treatment with the EETC inhibited RANKL mRNA expression but did not affect OPG mRNA expression in DPB-LPS–treated hFOB1.19 cells (Fig. 4c). Consequently, the increased RANKL/OPG ratio in the DPB-LPS–treated hFOB1.19 cells was remarkably decreased by treatment with EETC. Thus, bone destruction by DPB-LPS was caused by the cooperative stimulation of osteoclast formation in BMMs and of RANKL expression in osteoblasts. EETC could be an important means of preventing bone loss due to oral bacteria.

## Discussion

Inflammation is the biological response of tissues to infection with pathogens and cell damage [15]. When harmful stimuli cause an inflammatory response, the body’s innate immune system involves the phagocytic activation of leukocytes and macrophages, and the increased release of inflammatory cytokines. Uncontrolled inflammation leads to disease progression because of the excessive production of inflammatory cytokines [16]. DPB are a leading cause of inflammation in periodontal disease [17]. Untreated gingivitis readily progresses to periodontitis and leads to tissue destruction. Bacterial plaque elaborate various compounds that elicit an inflammatory response and lead to destruction of the gingival tissue, which may progress to destruction of the periodontal ligaments. Plaque control is required to prevent further progression of inflammation. In this study, we tried to elucidate the critical factors involved in plaque bacteria-induced inflammation and effective natural anti-inflammatory substances for plaque bacteria control.

The in vitro anti-bacterial activity of EETC against DPB was evaluated. Disc diffusion assay results showed that EETC effectively suppressed the DPB growth and the anti-bacterial activity of EETC were increased with increasing concentration of extract. This concentration of EETC was not cytotoxic to gingival epithelial cells, gingival fibroblasts and macrophage. The induction of inflammatory factors and proteases that contributed to the inflammatory reaction were also abolished by EETC. These results indicate that EETC could effectively suppress the inflammatory responses induced by plaque bacteria. The collective data indicate that EETC is useful in reducing bacterial plaque accumulation and gingival inflammation, thereby preventing periodontal disease. Since the efficacy and safety of EETC has been established, it can be developed into a mouthwash without added alcohol. Many commercially available mouthwashes contain alcohol, which increases the risk of oral cancers and dry mouth.

LPS is also known as lipoglycans and endotoxins. The molecules is a component of the outer membrane of the cell wall of gram-negative bacteria, and is one of the most powerful bacterial virulence factors in the pro-inflammatory response [18]. Initiation of the inflammatory response by LPS stimulates a strong immune response. LPS acts as the prototypical endotoxin through binding to the CD14/TLR4/MD2 receptor complex in many cell types, but especially in monocytes, dendritic cells, macrophages, and B cells, which promotes the secretion of pro-inflammatory cytokines, nitric oxide, and eicosanoids [19]. Therefore, LPS is a central molecule in the pathogenesis of certain bacterial infections [20]. In this study, *in vitro* co-culture with gingival epithelial cells and plaque bacteria caused pH changes of the culture medium during 24 h of incubation. Therefore, LPS was extracted from DPBs and its capacity to induce inflammation was verified. DPB-LPS significantly increased the levels of inflammatory factors in gingival epithelial cells, gingival fibroblasts and macrophages. EETC also effectively suppressed the levels of DPB-LPS-induced inflammatory factors.

EETC significantly suppressed growth of DPB and DPB-mediated periodontal disease. Antibiotics are used to both treat and prevent bacterial infections [21]. Antibiotics like penicillin and erythromycin used to be highly effective against many bacterial species and strains. They are now less effective because these organisms have developed increased resistance to the drugs [22]. Dental plaque, which adheres to the gingival sulcus, consists mainly of bacterial cells, salivary polymers, and bacterial extracellular products. Bacterial LPS stimulates the host cells, including macrophages, fibroblasts, and epithelial cells, which abundantly produce cytokines, thereby triggering a direct or an indirect immune response through inflammatory processes [23]. The growth of bacterial biofilms increases antibiotic resistance and has been implicated in the etiopathogenesis of gingivitis and periodontal disease by inducing inflammation.

An effective substance is needed that will prevent and treat bacterial plaque-mediated disease by controlling bacterial growth and the inflammatory reaction. The fruits of the *T. chebula*, which has been amply verified as being medicinally valuable, has been used to treat cancer, cardiovascular diseases, paralysis, leprosy, ulcers, gout, arthritis, epilepsy, cough, fever, diarrhea, gastroenteritis, skin disorders, urinary tract infection, and wound infections [10, 24]. *T. chebula* fruit is also effective in the treatment of bacterial infections [25, 26]. Clinical trials of *T. chebula* fruit extract as a mouthwash preparation have reported reduced plaque accumulation and gingival inflammation [27, 28]. The present findings provide mechanistic details for these beneficial effects.

Most dental care products exert their antibacterial effects by eliminating oral bacteria. Instead, LPS from other types of bacterial cells, such as *E. coli* and *Pseudomonas aeruginosa*, have been used to induce the inflammation. Inflammatory events related to DPB-derived endotoxins have not been evaluated as intensively [29, 30], and the value of anti-microbial dental care products to treat periodontal disease has not been sufficiently verified. Thus, there was a need to simultaneously investigate inflammatory events due to DPB-derived endotoxin and elucidate an effective anti-microbial component for periodontal disease. Here we demonstrate that DPB and DPB- significantly increase inflammation-associated elements such as COX-2 and PGE2. Many inflammatory molecules and proteases are also upregulated by DPB-LPS and lead to matrix damage. In addition, DPB-LPS stimulation promotes osteoclast formation, causing bone resorption. The risk of bone loss tends to increase in patients with inflammatory conditions [31]. Inflammatory cytokines modulate osteoblast and osteoclast activity [32]. Interleukins (IL), tumor necrosis factor (TNF), and tumor necrosis factor receptor superfamily members (TNFRSF) perturb bone homeostasis, leading to increased cartilage degradation and bone resorption by osteoclasts and inhibiting bone formation by osteoblasts [33].

In the present study, IL-3, IL-16, CXCL10, TNFRSF1A, TNFRSF1B, M-CSF 1, and CCL4 were increased in DPB-LPS-stimulated gingival epithelial cells. Proteases including MMP-3, MMP-7, KLK 10, CTSC, and PC9, which were also increased by DPB-LPS stimulation, might also contribute to the inflammatory reaction, thereby leading to matrix degradation and bone destruction. However, the levels of inflammation-related molecules and osteoclastic bone resorption were significantly reduced by treatment with EETC. EETC also exhibited anti-bacterial properties by arresting the growth of DPB. These findings suggest that the EETC could be used to treat DPB-mediated periodontal disease.

Osteoclast differentiation and bone resorption activity require stimulation by the receptor activator of nuclear factor-kappaB (RANKL) expressed on osteoblasts [34]. In the present study, increased RANKL mRNA expression was observed in DPB-LPS-stimulated hFOB1.19 human osteoblast cells. In addition, DPB-LPS stimulated osteoclast formation and bone resorption under RANKL-limited conditions. These results are consistent with the results of previous studies showing that LPS stimulated osteoclastogenesis [35–37]. These results indicate that DPB-LPS promotes functional osteoclast formation through osteoblast-dependent and osteoblast-independent pathways. DPB-LPS-induced osteoclast formation and bone resorption were also significantly abolished by the EETC treatment.

## Conclusions

In conclusion, treatment with the EETC inhibits the growth of DPB as well as DPB-induced inflammation, and effectively abolishes DPB-LPS-induced osteoclastic bone resorption in vitro. EETC is an effective botanical chemopreventive agent that can modulate DPB-induced inflammatory factors involved in gingivitis and periodontal disease. EETC can be considered a promising anti-bacterial and anti-oral inflammatory agent capable of preventing the development of gingivitis and periodontitis. Further research is needed to isolate and identify the beneficial chemical constituents in the extract that could be exploited for pharmaceutical use.

## Abbreviations

COX-2: Cyclooxygenase 2
LPS: Lipopolysaccharide
PGE2: Prostaglandin E2
RANKL: Receptor activator of nuclear factor κB ligand
*T. chebula*: *Terminalia chebula*
